# Parental Burnout—A Model of Risk Factors and Protective Resources Among Mothers of Children with/Without Special Needs

**DOI:** 10.3390/ejihpe14110189

**Published:** 2024-11-11

**Authors:** Yifat Findling, Michal Itzhaki, Sivia Barnoy

**Affiliations:** Department of Nursing, School of Health Professions, Faculty of Medical & Health Sciences, Tel Aviv University, Tel Aviv 6997801, Israel; itzhakim@tauex.tau.ac.il (M.I.); sivia@tauex.tau.ac.il (S.B.)

**Keywords:** parental burnout, mothers of children with special needs, caregiver burden, social support, emotion work, learned resourcefulness

## Abstract

Parents of children With Special Needs and Disabilities (W-SND) who require long-term healthcare are at high risk of Parental Burnout (PB). However, most studies have focused on PB among parents of children Without Special Needs (WO-SN). This study aimed to develop a new model explaining PB of mothers of children W-SND/WO-SN. The main hypothesis was that the nexus of correlations between risk factors of PB (severity of child’s disability/challenge, perceived caregiver burden) and protective resources (social support, learned resourcefulness, deep emotion work) will explain the variance of PB of mothers of children W-SND and WO-SN. A questionnaire assessing PB, its risk factors, and protective resources was completed by 352 Israeli mothers of children W-SND (mean age 36.9) or WO-SN (mean age 32.3). The child’s disabilities were communicative, physical, intellectual and developmental. The main results are that mothers of children W-SND reported higher PB, higher caregiver burden, and a higher severity of disability. About 50% of PB variance was significantly explained by the nexus of correlations between selected risk and protective factors. Among all mothers, the more social support they received, the higher their learned-resourcefulness. However, learned resourcefulness mediates the correlation between caregiver burden and PB among mothers of children W-SND. Accordingly, it is important to increase awareness among healthcare professionals regarding the risk factors and symptoms of PB, and to develop workshops on protective resources in order to prevent PB and promote mothers’ well-being. Further research should be conducted among fathers and parents from diverse cultures.

## 1. Introduction

Parenting includes raising and nurturing a child for many years, from infancy to adulthood, and requires constant responsibility [1,2]. It is one of the most rewarding experiences in human life and may involve much happiness and pleasure. However, parenting has its challenges, including stressful situations, which can be exhausting. Parenting children With Special Needs and Disabilities (W-SND) demands long-term care over and above routine parenting requirements. This may affect parents’ daily functioning due to the physical, emotional, economic, and social pressures [3,4]. Parents need to cope with their child’s continual treatments which can be highly stressful and increase the risk of Parental Burnout (PB) [5,6,7,8]. 

To date, most studies have focused on PB among parents of children Without Special Needs (WO-SN) or chronic illnesses, while few studies have conducted among parents (especially mothers) of children W-SND [3,9]. The significance of the current research is its novelty in studying parental burnout among mothers of children with special needs and disabilities; the focus of most studies conducted so far has been mainly among parents of children with specific chronic diseases, and thus their results do not enable generalization and explanation of the risk factors of PB among mothers of children W-SND.

The main aim of the study was to examine the fitness of a research model describing the contribution of the nexus of correlations between risk factors and protective resources of PB among mothers of children W-SND and WO-SN. The risk factors that the study focused on included the perceived severity of the child’s disability and caregiver burden, while protective resources included social support resources [10], learned resourcefulness as a cognitive resource that comprises a repertoire of skills that allow for self-regulation [11], and emotion work (EW) as an emotional resource which allows for the management and adaption of one’s emotions to social expectations [12].

The research was based on the Job Demands-Resources Model of Burnout (JD-R) [13], which claims that working conditions (physical, psychological, social, or organizational aspects of the job) include two main categories: (i) job demands that may cause stress and fatigue and positively predict job burnout (e.g., physical environment, task overload), and (ii) job resources that may reduce job demands, contribute to the improvement of functioning and the achievement of work goals, and can negatively predict job burnout (e.g., collegial support, autonomy in decision making). According to the JD-R model, maintaining a balance between job demands and job resources may reduce job burnout [13,14,15,16].

According to [17], the JD-R theoretical model [13] can be appropriately applied in the study of parental burnout, as parenting is considered a complex and stressful job with many demands. PB develops due to a chronic imbalance between demands (i.e., risk factors) and protective resources. Thus, the risk for PB is higher when the parent’s protective resources are insufficient to cope with the demands of their parental role. However, differences exist between job burnout and PB, i.e., parents cannot quit their role while a worker can quit and be replaced by another, and unlike in regular jobs, boundaries between personal and professional areas are unclear because parenting demands are ongoing [18,19]. 

### 1.1. Parental Burnout

PB is a specific syndrome accompanied by chronic exhaustion: physical, mental, emotional, and cognitive, related to and resulting from the roles of the parent [20]. It affects parents worldwide, with an estimated 8% across various cultures, particularly in Western countries [21]. PB includes four dimensions: physical and emotional exhaustion expressed in chronic fatigue; emotional distance from the child which makes it difficult for the parent to care for them beyond their basic needs; saturation from the role of the parent that results in an inability to cope and enjoy being a parent; and parental self-contrast which stems from dissatisfaction with being the parent in the present compared to the past [20,22]. PB develops mainly due to an imbalance between risk factors (ongoing exposure to parenting demands) and protective resources (the type and quantity of coping resources available to the parent). Parents can utilize protective resources against PB, such as high emotional intelligence, self-compassion, positive thinking, appropriate management of leisure time, high parental skills, and environmental and societal support [20,23]. Conversely, parents have a higher risk of PB if they attempt perfectionism in parenting [24] (Kawamoto et al., 2018), are prone to neuroticism, i.e., emotional imbalance [25], have low emotional intelligence, tend to pessimistic thinking, lack child-rearing skills, and lack social and emotional support [20]. As a result of PB, parents may become more vulnerable, emotionally distant from their children, their functioning is merely practical, and their interaction with their children may be limited to instrumental aspects only [26,27,28,29].

### 1.2. Risk Factors for Parental Burnout

The research model focused on the risk factors of perceived caregiver burden and perceived severity of disability.

#### 1.2.1. Perceived Caregiver Burden as a Risk Factor

An informal caregiver’s ‘burden’ may be expressed in an emotional response to caring for a family member. It is perceived as a response to prolonged multidimensional pressures that include physical, psychological, emotional, social, and economic dimensions related to the therapeutic needs of the family member with the disability [25,30]. This prolonged coping has negative consequences for the caregiver and is accompanied by high stress levels related to their subjective perception of the burden that may affect their quality of life [4]. In many cultures, women are traditionally entrusted with childcare, even when working, and thus they are more exposed to additional parental stress, which raises their risk of parental burnout [21,31]. Additionally, during situations of national emergencies (such as a pandemic lockdown or an ongoing war), families’ economic situation tends to worsen, placing an extra burden on women to financially support their families in addition to the traditional maternal roles, and thus enhancing the risk of PB [32]. Studies conducted on informal caregivers have shown that the treatment burden tends to negatively affect family relationships, reduce family activities, and harm the parents’ marriage [33]. Studies have shown that parents of children with chronic diseases are at higher risk of PB than parents of healthy children, in addition to an increased perceived caregiver burden, mainly due to the prolonged care of the child, the impairment of normative family life management, and neglect of their physical and emotional needs [6,7,25,34]. Studies conducted so far on the relationship between care burden and parental burnout have focused mainly on parents of children with specific chronic diseases, which does not allow generalization and explanation on factors related to PB among parents of children with special needs.

#### 1.2.2. Perceived Caregiver Burden Due to the Perceived Severity of the Disability

Caregiver burden of treatment is strongly affected by the perception of the severity of the disability in terms of the type of disability and the child’s level of functioning and independence [35]. Accordingly, disability severity is related to the difficulty involved in fulfilling daily tasks related to the child, which contributes to caregiver burden, and may gradually lead to the development of PB [36].

### 1.3. Protective Resources for Coping with Parental Burnout

The research model focused on three protective resources: social support, learned resourcefulness, and emotion work.

#### 1.3.1. Social Support—A Social Resource

Social support is an exchange of resources, information, and assistance between at least two people, from official and unofficial sources, to maintain one’s well-being [37]. According to Hobfoll’s Conservation of Resources Theory [10], people are motivated throughout their life to hold, preserve and protect their personal resources. Social support is perceived as an important resource, as it provides or facilitates the preservation of other valued coping resources. Thus, while coping with ongoing stress, social support enables the preservation of all available resources together with one’s personal resources.

Most studies conducted so far on the relationship between social support and burnout have focused on burnout among workers who provide services (e.g., teaching and nursing). In these studies, social support was found to be a significant coping resource that reduces the development of burnout, especially in the dimension of physical and emotional exhaustion [38,39,40]. However, several studies have examined the relationship between social support and burnout among parents in general and parents of children with disabilities in particular. These studies revealed that social support from family, neighbors, and friends, is a significant protective factor that helps parents cope with PB since it provides help in daily tasks related to caring for the child and gives a sense of partnership in their difficulties. Thus, lack of social support is a risk factor for the development of PB [20,41,42].

#### 1.3.2. Learned Resourcefulness—A Cognitive-Behavioral Resource

Learned resourcefulness is a cognitive-behavioral resource that includes a repertoire of skills with which a person can regulate internal events (e.g., pain, emotions, unwanted thoughts, anxiety, and depression) to achieve better control over the effects these events might have on their behavior [11]. Resourcefulness can be learned, i.e., people can be taught how to acquire self-control skills, regulate unpleasant emotional experiences, plan steps to achieve goals, and learn systematic ways for problem solving.

Previous studies have demonstrated the moderating relationship of a high level of learned resourcefulness between caregiver burden and burnout due to high levels of self-control [43,44,45].

In most studies, social support and learned resourcefulness are described as two separate resources that help individuals cope with stress, perceived caregiver burden, depression, and loneliness. Both social support and learned resourcefulness have each been found to predict a good quality of life, positive self-esteem, high self-confidence, and adaptive functioning. These allow people to be less worried, depressed, or frustrated [46,47,48]. 

#### 1.3.3. Emotion Work—An Emotional Resource

While performing EW, people try to manage and adapt their feelings to the emotions that others expect them to feel in social situations by arousing desirable (positive) emotions or suppressing unwanted (negative) emotions [12,49]. Usually, EW is an unconscious process that may become conscious when the individual senses an emotional gap between their experienced and expected emotions. EW aims to create a real change in an individual’s feelings that can be learned. It is carried out using four techniques that can coexist or be performed separately: cognitive, physical, expressive, and superficial [49,50]. Studies examining EW have mostly been conducted among service providers and have found a direct correlation between the ability to perform EW and level of burnout at work [51,52,53]. Additionally, some studies have investigated EW among health staff (physicians, nurses) [54] and found a negative correlation between the superficial EW technique (surface acting) that enhances the displayed (but not sincerely experienced) emotions and sufficient knowledge to provide patient care safely. As a protective resource that can be learned, deep EW may help cope with PB. In the context of the current study, studies among unofficial caregivers of family members with chronic disabilities found that deep EW helps reduce burnout resulting from a perceived caregiver burden. Conversely, failure to manage their emotions might create stress which increases their caregiver burden [55,56]. 

### 1.4. Model of Risk Factors and Protective Resources of Parental-Burnout 

**Figure 1:**
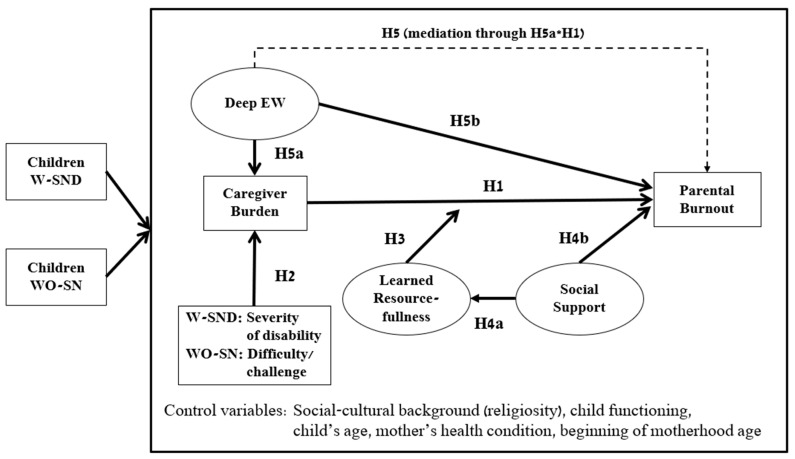
Model of Risk Factors and Protective Resources of Parental Burnout.

Based on the reviewed literature, the research model is presented in Figure 1.

### 1.5. Research Hypotheses

**H1:**  *The higher the perceived caregiver burden, the mother’s level of PB will be found higher.*

**H2:**  *The more the disability (W-SND)/challenge/difficulty (WO-SN) is perceived as severe, the heavier the perceived caregiver burden will be.*

**H3:**  *Learned resourcefulness will moderate the correlation between perceived caregiver burden and PB; among mothers with high levels of learned resourcefulness, the correlation between the perceived caregiver burden and PB will be weaker, and vice versa.*

**H4:**  *T* *he more social support mothers receive, (a) their learned resourcefulness will be found higher and (b) their PB will be found lower.*

**H5a:**  *Caregiver burden will mediate the correlation between deep EW and PB.*

**H5b:**  *The nexus of correlations between risk factors and protective resources will explain the variance of PB of mothers of children W-SND and WO-SN.*

## 2. Methods

### 2.1. Design

This current research was a quantitative descriptive, cross-sectional study utilizing self-report validated questionnaires. A pilot study was carried out before the final research [9] to test the reliability and validity of the research questionnaires and to adjust them to the research sample (mothers of children with or without special needs).

### 2.2. Participants

The sample included 352 mothers, 50% (N = 176) of whom have a child W-SND treated in one of three child development centers, and 50% (N = 176) with a child WO-SN treated in one of three mother–child health centers in Israel (religious and non-religious).

Mothers who expressed consent to participate in the study were included in the study. The mothers were selected through a non-probability convenience sampling procedure. The criteria for inclusion in the research were mothers without physical or cognitive disabilities who were aged 20–55 years. The mothers of children W-SND have only one child W-SND aged 1.5–5 years.

### 2.3. Procedure

Between 2021 and 2022, nurses and secretaries (who received guidance from the researchers) working in three mother–child health centers and in three child development centers distributed the questionnaires in anonymous sealed envelopes to mothers of children W-SND and WO-SN.

Mothers who agreed to participate were given an oral and written explanation about the study and anonymity was guaranteed. We ensured that the health staff received explanations and directions in order to guarantee minimal bias. There was an 88% response rate for completion of the questionnaires.

### 2.4. Instruments

#### 2.4.1. Social, Emotional, and Practical Support Questionnaire

The questionnaire measures social, emotional, and practical support received from significant people (nuclear and/or extended family, friends, neighbors, and health professionals (nurses/doctors) (constructed in Hebrew by London [57]). The questionnaire included 12 items, for example, “To what extent do you feel that you receive practical support from friends?”. The answers were rated on a scale ranging between 1 (insufficient support) to 5 (sufficient support). The social support score was calculated as the average of the 12 questions. In the present study, this questionnaire demonstrated high internal reliability among all mothers of children W-SND and WO-SN (α = 0.870).

#### 2.4.2. Learned Resourcefulness Questionnaire

The questionnaire examines the extent to which the individual applies self-control skills to reduce unwanted effects on their behavior (constructed in Hebrew by Rosenbaum [58]). The original questionnaire was shortened in the pilot study from 36 items and included 17 statements that related directly to parenting [9]. For example, “While an unpleasant thought bothers me, I try to think about pleasant things”. The answers were rated on a scale ranging between 1 (very uncharacteristic of me) to 6 (very characteristic of me). The learned resourcefulness score was calculated as the average of the 17 statements. Its internal reliability was high among all mothers of children W-SND and WO-SN in the pilot study (N = 98, α = 0.849), and in the final study (N = 352, α = 0.878).

#### 2.4.3. Caregiver Burden of Treatment Questionnaire

The questionnaire measures the caregiver’s burden of treatment [59,60]. It includes 12 items, for example, “Due to the time spent with your child, you don’t have enough time left for yourself”. The items were ranked on a scale ranging from 1 (never, i.e., a low level of caregiver’s burden of treatment) to 7 (daily, i.e., a high level of caregiver’s burden of treatment). The caregiver’s burden of treatment score was the average of the responses to the 12 items. The internal reliability was high in both previous research (α = 0.830 [61]; α = 0.880 [62]) and in the current study (α = 0.842).

#### 2.4.4. Emotion Work Questionnaire

The questionnaire examines the extent to which mothers of children perform EW while caring for their child, based on Hochschild’s [12] theory and a research questionnaire on the EW and resilience of nursing and medical staff while treating children from the Palestinian Authority hospitalized in Israel [63]. It was adjusted to the population of mothers of children W-SND and WO-SN as caregivers in the pilot study. The deep EW scale measured three techniques (physical, expressive, cognitive) and included ten items, for example, “I remind myself that my life includes additional activities in order to feel happy”. The items were ranked on a scale ranging from 1 (do not agree at all) to 5 (agree to a large extent). The EW score was the average of the responses to the ten items. In previous research (α = 0.930 [63]) and in the current study (α = 0.880), the reliability was found to be high.

#### 2.4.5. The Parental Burnout Assessment

The Parental Burnout Assessment (PBA) [17] questionnaire includes 23 items. The questionnaire was adjusted to the current research by changing the word “parent” to “mother”. The PBA scale includes four PB dimensions: Physical and emotional exhaustion in the parental role, nine items (e.g., “I’m so tired out by my role as a mother that sleeping doesn’t seem like enough”); emotional distancing from the child, three items (e.g., “Outside the usual routines (bedtime, meals), I’m no longer able to make an effort for my child(ren)”; saturation from the parental role, five items (e.g., “I feel like I can’t cope as a mother”); and contrast with previous parental-self, six items (e.g., “I don’t think I’m the good mother that I used to be to my child(ren)”). Mothers were asked to indicate how often they felt in the past year each feeling on a scale ranging from 1 = never, 2 = a few times a year, 3 = once a month or less, 4 = as few times a month, 5 = once a week, 6 = a few times a week, 7 = every day. The mean PB was the average of the responses to the 23 items. The sum of all the responses to the items was also calculated (range 23–161 [23x1–23x7]). The risk of PB was categorized according to Roskam et al. [17]: 0 = “no PB” (<35), 1 = “low risk” (36–53), 2 = “medium risk” (54–70), 3 = “high risk” (71–88), and 4 = “has PB” (89+). In the current study, the reliability of the questionnaire was high α = 0.95.

#### 2.4.6. Caregiver’s Perceived Level of Child’s Disability or Challenge/Difficulty in Raising Their Child

The mothers of children W-SND were requested to define their perception of the severity of their child’s daily functional disability (“How would you define the level of severity of your child’s disability, regarding daily functioning?”), and mothers of children WO-SN were asked whether they found that raising their child is challenging or difficult (“how would you define the level of challenge/difficulty raising your child, regarding daily functioning?”). Their responses were ranked on a five-rank scale, between 1 (very mild) and 5 (very severe).

#### 2.4.7. WeeFim—Functional Independence Measure for Children

The questionnaire assesses the functioning of healthy children from six months to seven years of age and in children with disabilities from six months to 12 years [64]. The WeeFim questionnaire is based on the Functional Independence Measure (FIM) scale for adults and was developed for children by Braun et al. [65]. The questionnaire includes 18 items describing the child’s functional ability in six areas that relate to the performance of the child’s daily activities: self-care, control of urine sphincters, transitions, mobility, communication and social cognition. Thirteen items belong to the motor dimension (e.g., “to what extent is you child able to eat by himself”) and five items to the cognitive dimension (e.g., “to what extent is you child able to express himself”). The items were rated on a Likert scale ranging between 1 (full dependence on another person’s help, lack of independence) to 7 (complete independence in performing the functional task, without the need for the assistance of another person). The final score was calculated as the standardized average of the items. The reliability of the child’s functioning questionnaire in the present study was high (α = 0.96).

#### 2.4.8. Background Characteristics

Background characteristics included religiosity, age, age when becoming a mother (mother’s age minus the firstborn child’s age), mother’s health condition in the past month (1 = not good to 5 = very good), child’s age and gender, and number of children. Mothers of children W-SND were asked the nature of their child’s impairment (cognitive developmental, physical, visual, hearing, communicative (autistic-spectrum disorders), chronic disease, or other).

### 2.5. Data Analyses

The data were processed using SPSS for Windows version 27. The reliability of the questionnaires was examined using Cronbach alpha (α). Descriptive statistics included frequencies, percentages, means, and standard deviations. The inference statistics included a comparison between mothers of children W-SND or WO-SN, using χ^2^ tests and independent samples *t*-tests. The effect size of the differences between means among mothers of children W-SND or WO-SN, was calculated by Cohen’s d (size effects around 0.20 are considered weak, around 0.50—moderate and above 0.80—strong [66,67]). Confirmatory factor analysis was used to calibrate the research values and confirm the validity of their construct. The hypotheses drawn from the theoretical model were tested using structural equation modeling (SEM) [68] using Mplus software version 8.9.

### 2.6. Ethical Considerations

The study was approved by the Helsinki committee of “Meuhedet Health Services” Clinics (approval #02290120) and by the ethics committee of Tel-Aviv University (approval #0001222-2). According to the Helsinki Committee approval, informed consent was accepted by the mothers’ agreement to complete the questionnaire.

## 3. Results

### 3.1. Sample Characteristics

The average age of the mothers of children W-SND (36.90 ± 5.63) was found to be higher than the mothers of children WO-SN (32.27 ± 5.88) (t = −7.53, *p* ≤ 0.001). Mothers of children W-SND became mothers at an older age (26.17 ± 4.97) than mothers of children WO-SN (24.33 ± 4.45) (t = −3.67, *p* ≤ 0.001). The average number of children in the family was similar (W-SND—4.22 ± 2.23) WO-SN (3.97 ± 2.62) (t = −0.941, *p* = 0.347). Mothers of children W-SND reported that their health condition in the past month (3.89 ± 0.68) was not as good as the mothers of children WO-SN (4.40 ± 0.60) (t =7.56, *p* ≤ 0.001). Most of the children were males (71%). The average age of the children W-SND (4.06 ± 0.97) was higher than children WO-SN (3.03 ± 1.09) (t = −9.35, *p* ≤ 0.001). The child’s disabilities were communicative (44.9%), physical (30.1%), intellectual developmental (27.8%), sensory vision (6.8%), sensory hearing (5.1%), and mental (2.8%). Some of the children had more than one disability. Most mothers (85.8%) reported that their child’s impairment did not have any known genetic component.

### 3.2. Parental Burnout of Mothers of Children W-SND/WO-SN

T-tests between groups were computed to test the differences in PB and its dimensions between mothers of children W-SND/WO-SN (Table 1).

According to Table 1, the overall level of PB (and its four dimensions) was found to be relatively low, but higher among mothers of children W-SND (2.12 ± 0.95) than among mothers of children WO-SN (1.48 ± 0.58) (t = −7.80, *p* ≤ 0.001). Thus, in both groups, a similar pattern was found in all four dimensions of PB, but the largest gap was found in the physical and emotional exhaustion dimension. The highest, yet moderate, level of PB in the physical and emotional exhaustion dimension was reported by mothers of children W-SND (2.55 ± 1.19) as compared to lower levels of PB in the other three dimensions.

A high risk of PB was found among six (3.4%) mothers of children W-SND, as compared to three (1.7%) mothers of children WO-SN. PB was found among seven (4%) mothers of children W-SND, as compared to one mother (0.6%) of children WO-SN. Conversely, 67 (38.1%) mothers of children W-SND had no risk of PB compared to 133 (75.6%) mothers of children WO-SN.

T-tests between groups were computed to test the differences between mothers of children W-SND/WO-SN in the risk factors and the protective resources of PB (Table 2).

According to Table 2, mothers of children W-SND perceived a higher caregiver burden than those of children WO-SN (2.77 ± 1.07 vs. 2.15 ± 0.91; t = −5.79, p ≤ 0.001). Mothers of children W-SND also reported less learned resourcefulness and performed deeper EW. Both groups of mothers similarly assessed their child’s functioning and their social support.

#### 3.2.1. Risk Factors for PB

Perceived caregiver burden of treatment (range 1–7). Mothers of children WO-SN reported a lower caregiver burden of treatment (2.15 ± 0.91) than mothers of children W-SND (2.77 ± 1.07) (t = −5.79, *p* ≤ 0.001). No significant difference was found in child functioning (t = −0.24, *p* = 0.086). In addition, according to the reported background variables, mothers of children W-SND perceived the severity of their child’s disability as moderate (2.94 ± 0.92). Additionally, 7.4% of these mothers reported that they perceive their child’s disability as not severe, as compared to 22.7% that perceived it as severe. Conversely, 76% of mothers of children WO-SN reported that they do not have any challenges/difficulties in raising their children. A high level (but not the highest) of challenge/difficulty in raising the child was reported by 15.3% of the mothers.

#### 3.2.2. Protective Resources

Social support (range 1–5): mothers of children WO-SN (2.60 ± 0.91) reported a moderate level of social support, but slightly higher than mothers of children W-SND (2.53 ± 0.91) (t = 0.74, *p* = 0.470). Learned resourcefulness (range 1–6): mothers of children WO-SN (4.36 ± 0.77) reported a slightly higher level of learned resourcefulness than mothers of children W-SND (4.14 ± 0.69) (t = 2.77, *p* = 0.035). Emotion work (range 1–5): mothers of children WO-SN (2.39 ± 0.93) reported a slightly lower level of deep EW than mothers of children W-SND (2.99 ± 0.72) (t = −6.77, *p* ≤ 0.001).

### 3.3. Hypothesis Testing

**Figure 2:**
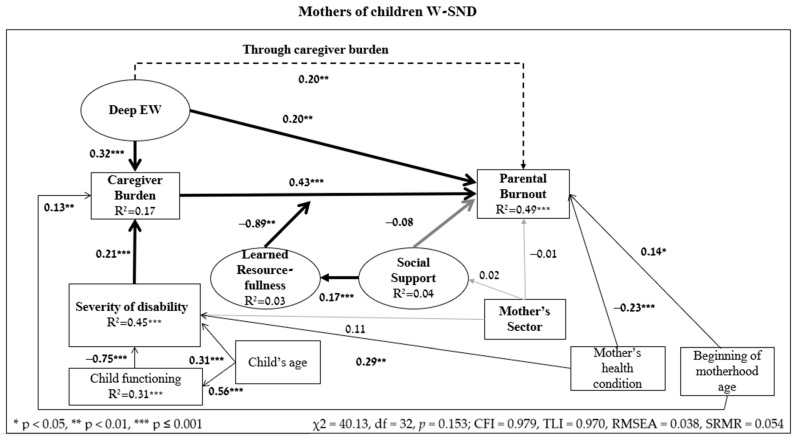
Results of SEM hypothesis testing among mothers of children W-SND (β standardized coefficients) (N = 176).

**Figure 3:**
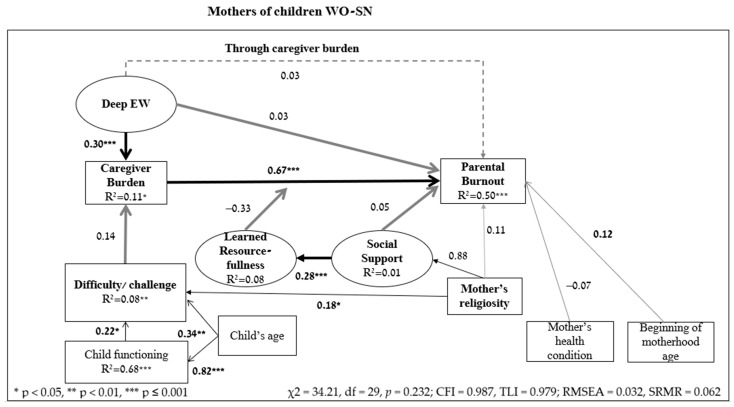
Results of SEM hypothesis testing among mothers of children WO-SN (β standardized coefficients) (N = 176).

In order to test the research hypothesis according to the theoretical model, SEM analysis was computed. The results are presented in Figure 2 (W-SND) and Figure 3 (WO-SN).

No significant effect was found for religiosity on PB. However, religious mothers of children WO-SN reported less challenge/difficulty raising their child (β = 0.18, *p* = 0.014) and received more social support (β = 0.88, *p* ≤ 0.001) than non-religious mothers. No similar effect was found among mothers of children W-SND. Among all mothers, as the age of the child increases, the mothers’ perception of the child’s functioning level is higher (W-SND—β = 0.56, *p* ≤ 0.001, WO-SN—β = 0.82, *p* ≤ 0.001). It was also found that the younger mothers of children W-SND became mothers, their perceived caregiver burden (β = 0.13, *p* = 0.007) and PB (β = 0.14, *p* = 0.013) were reported higher. No similar effect was found among mothers of children WO-SN. Additionally, the mothers of children W-SND who reported more positively on their own health condition in the past month, perceived their child’s disability as less severe (β = 0.29, *p* ≤ 0.001) and reported lower PB (β= −0.23, *p* ≤ 0.001). No similar effect was found among mothers of children WO-SN.

According to the results of the model’s hypothesis testing, Hypothesis 1 was confirmed: the higher the mothers perceived their caregiver burden, the higher their level of PB (W-SND β = 0.43, *p* ≤ 0.001; WO-SN β = 0.67, *p* ≤ 0.001).

Hypothesis 2 stated that the more the disability/challenge/difficulty was perceived as severe, the heavier the perceived caregiver burden. Hypothesis 2 was confirmed among mothers of children W-SND (β = 0.21, *p* ≤ 0.001) but not among mothers of children WO-SN (β = 0.14, *p* = 0.059).

Hypothesis 3 assumed that the correlation between perceived caregiver burden and PB was moderated by learned resourcefulness. Among mothers of children W-SND, the fit indices of the model, including the moderation effect, were found to be non-significant (χ^2^ = 9.86, df = 5, *p* = 0.079; χ^2^/df = 1.97; CFI = 0.970, TLI = 0.928; RMSEA = 0.074, SRMR = 0.046), i.e., the empirical data matched the hypothesis model. Hypothesis 3 was confirmed for mothers of children W-SND (β = −0.89, *p* = 0.008) such that among those with low levels of learned resourcefulness, the correlation between perceived caregiver burden and PB was stronger; whereas among those with high levels of learned resourcefulness, this correlation was weaker. Among mothers of children WO-SN, the fit indices of the model were found to be insignificant (χ^2^ = 5.55, df = 4, *p* = 0.235; CFI = 0.990, TLI = 0.971; RMSEA = 0.047, SRMR = 0.044); i.e., the empirical data matched the hypothesis model. However, the moderation hypothesis was not confirmed (β = −0.33, *p* = 0.200).

Hypothesis 4 tested the direct correlations between social support, learned resourcefulness, and PB. Hypothesis 4a was confirmed among mothers of children W-SND (β = 0.17, *p* = 0.019) and WO-SN (β = 0.28, *p* ≤ 0.001) such that the more social support the mothers received, the higher their learned resourcefulness. According to Hypothesis 4b, the more social support mothers receive, the lower their PB. This hypothesis was not confirmed (W-SND β = −0.08, *p* = 0.126; WO-SN β = 0.05, *p* = 0.449).

According to Hypothesis 5, the perceived caregiver burden mediated the correlation between EW and PB. This hypothesis was confirmed among mothers of children W-SND (indirect = 0.135, *p* ≤ 0.001, 95%CI [0.07, 0.22]) and WO-SN (indirect = 0.199, *p* ≤ 0.001, 95%CI [0.11, 0.30]). Additionally, among mothers of children W-SND (β = 0.20, *p* ≤ 0.001) there was also a direct correlation between EW and PB (and not only indirectly through perceived caregiver burden), i.e., a partial mediation. However, among mothers of children WO-SN, the correlation between EW and PB was only indirect through perceived caregiver burden (β = 0.03, *p* = 0.551), i.e., full mediation.

Hypothesis 6 that tested the research model was confirmed, such that the correlations between risk factors and protective resources explained the variance of PB of mothers of children W-SND/WO-SN. It was found that the correlations significantly explained the variance of PB among mothers of children W-SND (R^2^ = 0.49, *p* ≤ 0.001) and WO-SN (R^2^ = 0.50, *p* ≤ 0.001). According to Figure 2 and Figure 3, among both groups of mothers, the empirical data matched the hypothesis model (W-SND χ^2^ = 40.13, df = 32, *p* = 0.153; CFI = 0.979, TLI = 0.970; RMSEA = 0.038, SRMR = 0.054; WO-SN χ^2^ = 34.21, df = 29, *p* = 0.232; CFI = 0.987, TLI = 0.979; RMSEA = 0.032, SRMR = 0.062).

Comparing the model fit among each group of mothers revealed that a significant difference was found between the model’s fit for mothers of children W-SND (χ^2^ = 30.00) and for mothers of children WO-SN (χ^2^ = 13.19). Namely, the correlations between protective resources and PB were found stronger among mothers of children W-SND (social support: d= −0.17, *p* = 0.015; learned resourcefulness: d = −0.24, *p* = 0.008; EW: d = 0.28, *p* = 0.002). This is despite the fact that the burden of treatment (as a risk factor) was found to have a similar effect on PB in both groups (d = 0.01, *p* = 0.913). In addition, similar correlations were found between EW (d = −0.10, *p* = 0.220) and perceived disability/challenge/difficulty (d = 0.15, *p* = 0.137) with caregiver burden, and between social support and learned resourcefulness (d = 0.18, *p* = 0.156).

## 4. Discussion

The ongoing care of children W-SND with chronic disabilities may impose a unique caregiver burden and affect parents’ daily functioning and relationships with their children. The parents’ perceived caregiver burden may lead to the development of PB symptoms, mainly due to an imbalance between risk factors for PB and available protective resources. The current study focuses on the phenomenon of PB among mothers of children W-SND.

### 4.1. The Phenomenon of PB Among Mothers of Children W-SND/WO-SN

It was found that the levels of PB and its dimensions were relatively low, but higher among mothers of children W-SND. The PB dimension of physical and emotional exhaustion in the parental role among mothers of children W-SND was found to have the highest self-reported rating. This finding may indicate the main effect of PB on the mothers of children W-SND. In accordance with these findings, previous studies have shown that parents of children with chronic diseases are at higher risk of PB than parents of healthy children, mainly because they are required to cope with many concurrent pressures including family, economic, emotional, and social demands among others, in addition to their child’s ongoing therapeutic requirements [6,7,69]. Moreover, the dimension of physical and emotional exhaustion may be the basis for parental optimal functioning [20] in addition to the other dimensions of PB.

### 4.2. The Relationship Between Risk Factors and Protective Resources for PB

The relationship between caregiver burden and PB. According to Hypothesis 1, it was found that the higher the mothers perceived their caregiver burden, the higher the PB. This finding can be explained on the basis of the fact that usually mothers are considered the primary caregivers of their children in general, and unofficial caregivers of their children W-SND [8]. The literature also describes the daily time-consuming demands that mothers of children W-SND experience in addition to their other roles and responsibilities which may lead to a high perceived caregiver burden [25,30]. These include finding different treatment options for their child’s special needs, locating suitable treatment professionals, finding an appropriate educational framework, scheduling evaluations and meetings with relevant therapists, participating in therapeutic sessions with the child, and continuously monitoring the child’s progress [70,71,72]. Moreover, mothers often have to deal with a complex reality to ensure that the needs of the child with disabilities do not overshadow the needs of her other children and their spouse [8,73]. The perceive caregiver burden is especially true for parents who strive for perfectionism in performing these multiple tasks [24] such that the burden may adversely affect their physical and emotional health [74,75]. To date, in regard to the health system in Israel, there are not enough organizations and programs that offer effective support to these mothers.

Hypothesis 2 was confirmed, such that the higher mothers of children W-SND perceived their child’s disability severity, the higher they perceived their caregiver burden. This finding is supported by previous studies which showed that when parents perceive their child’s disability as more severe and their functional ability as lower, they report more burden and difficulty in caring for their child’s special needs [35,36,76].

In addition to the aforementioned risk factors for PB, the study investigated three protective resources that may reduce PB. It was found that for mothers of children W-SND, the cognitive coping resource of learned resourcefulness moderated the relationship between their perceived caregiver burden and PB (confirming Hypothesis 3). This means that among mothers with a high level of learned resourcefulness, the relationship between the perceived caregiver burden and PB was weaker, while among mothers with a low level of learned resourcefulness, this relationship was found to be stronger. The implication of this finding is that learned resourcefulness can reduce the effect of perceived caregiver burden on PB. According to the literature, learning how to be resourceful may enable mothers to acquire a better capacity for self-control over their negative emotions, help them effectively regulate and manage stress, cope with challenging circumstances, and make decisions in their own and their families’ best interest [11].

It was also found that the more social support mothers receive, their level of learned resourcefulness was found higher (confirming Hypothesis 4a). It is possible that social support provides mothers with the encouragement, motivation, and resources they need to develop ways of coping with difficult situations and challenges, contributing to their ability to develop learned resourcefulness as a protective resource. Similarly, social support, adaptation to daily concerns, and the ability to use positive patterns of thought significantly predicted learned resourcefulness among African American mothers who are considered primary caregivers of their children [77]. However, the lack of social support per se may serve as a risk factor for PB [20,41,42].

Contrary to Hypothesis 4b, it was found that receiving social support was unrelated to the PB level. A possible explanation for this lack of correlation is the nature of the social support, its quality, as well as the timing of when it is provided. All these are crucial for effectively assisting mothers to cope with stressors and challenges, and as a result, may reduce the risk of PB. For example, social support focused on problem solving and practical assistance may be more effective in reducing PB compared to friends’ support that usually has good intentions but can only offer general advice that is not necessarily appropriate for the needs and circumstances of mothers of children W-SND [78,79]. It is also possible that mothers do use social support to cope with the symptoms of PB, but do not simultaneously address the causes of the factors that led to the development of their PB. The latter include personality tendencies (tendency to pessimistic thought patterns, parental perfectionism), lack of parental skills for raising children, and low cooperation in parenthood [20]. In such cases, the support of friends and family may provide some temporary relief, but it will not necessarily lead to a reduction in long-term PB [80]. The timing of social support is also important; at times that mothers feel emotionally and consciously overwhelmed, they find it difficult to benefit from the social support offered to them, even though it may help reduce their risk of PB [81].

Another coping resource against PB is deep EW. Our findings indicate that perceived caregiver burden mediates the correlation between deep EW and PB among all mothers (confirming Hypothesis 5). Additionally, among mothers of children W-SND only, a direct correlation between EW and PB was found (i.e., a partial mediation in addition to the indirect effect through perceived caregiver burden). Among mothers of children WO-SN, the correlation between EW and PB was only indirect (i.e., fully mediated through perceived caregiver burden), as hypothesized. The meaning of these findings is that performing deep EW serves as an effective protective resource against PB among mothers of children W-SND because its effect on reducing PB is both direct (disregarding the perceived level of caregiver burden of treatment) and indirect (considering the perceived caregiver burden of treatment). In comparison, among mothers of children WO-SN, deep EW is effective only when the mothers experience a high caregiver burden of treatment. These findings imply that deep EW should be an inherent part of interventions whereby mothers of children W-SND are taught the physical, expressive, and cognitive techniques of deep EW.

The study found that the correlation between deep EW and PB is positive among mothers of children W-SND. This may imply that mothers of children W-SND in particular often perform EW that may lead them to feel overloaded, stressed, and exhausted. This is supported in the literature where it is described that mothers of children W-SND sometimes need to find ways to sensibly manage their emotions and even hide their feelings (e.g., stress, sadness, or frustration from their environment); a process that might increase rather than reduce their PB [82,83]. However, it is important to note that although EW can be demanding and challenging in itself and may even lead to an increase in the perceived caregiver burden and risk of PB, deep EW can ultimately serve as a protective resource with the potential for mothers to be able cope with complex parenting for their child W-SND and ultimately may reduce PB.

Finally, the nexus of correlations between the model’s risk factors and protective resources significantly explain about half of the variance in PB among mothers of children W-SND/WO-SN, confirming Hypothesis 6. Additionally, this relationship was significantly stronger among mothers of children W-SND. The findings support the importance of combining within one model [13,14] the protective resources for coping with PB, according to the three theories of social [10], cognitive [11], and emotional [12,49,50] resources.

### 4.3. Limitations of the Study and Recommendations for Further Research

In the current research mothers of children with specific disabilities may have had an unproportional impact on the results. Therefore, we suggest future investigations to sample groups of mothers of children with additional disabilities that experience other types and levels of difficulty. We also recommended to conduct additional studies among a variety of social and cultural backgrounds.

As parents of children W-SND face unique personal, marital and social challenges, further research should examine unique risk factors related to their condition. For example, socioeconomic status (low income or low levels of education may be related to financial stress or limited access to support services), lack of a supportive community and access to available support services, social or policy-related aspects (including financial assistance or counseling when needed).

Regarding learned resourcefulness as a protective resource, we suggest classifying the mothers into four distinct groups (W-SND/WO-SN X high/low levels of learned resourcefulness) and reassessing the research model after establishing the theoretical basis for this distinction and producing additional hypotheses.

It is also recommended to conduct similar research, using the “Model of Risk Factors and Protective Resources of Parental-Burnout” among fathers of children W-SND/WO-SN and among parents as dyads. These findings may contribute to the planning and development of programs for the entire family. For example, such a program could focus on balancing between needs, general tasks, and tasks involved in caring for the child, as well as the contribution of each family member to the required treatment.

## 5. Summary and Conclusions

This research examined a model of relationships between risk factors for PB and protective resources, that may alleviate burnout and balance the demands from mothers regarding their perceived caregiver burden [13,14,22]. 

The theoretical innovation of this research model lies in establishing the importance of combining the three theories of social, cognitive, and emotional protective resources, for coping with PB and reducing it in order to promote mothers’ well-being.

The first conclusion leads to the need to develop an intervention program for mothers of children W-SND, in which emphasis is placed on balancing their child’s needs alongside their personal needs. The goal of such a program is to reduce the risk factors for PB and to provide and strengthen their protective resources, including those investigated in this study (social support, learned resourcefulness, and emotion work). It is also important to identify the level of PB of these mothers using the PB assessment used in this study in addition to family and environmental reports, and paying attention to symptoms of exhaustion, depression, anxiety, and self-neglect. Accordingly, the multidisciplinary health team should be given appropriate tools for identifying mothers’ levels of PB.

We also suggest developing awareness in nursing studies and developing educational management practice regarding PB. Nursing students as well as healthcare staff should be guided to observe certain symptoms of the child resulting from the mother failing to provide essential treatments, signs of neglect and/or abuse, and receive clear instructions for offering these mothers appropriate professional interventions.

In summary, PB is explained by the nexus of correlations between risk factors and protective resources, beyond the effect of the child’s functioning, the mother’s perception of severity of disability, sense of caregiver burden, and the child’s dependency upon her.

## Figures and Tables

**Table 1:** Distribution of PB dimensions by group (W-SND/WO-SN).

	Overall Parental Burnout	Physical and Emotional Exhaustion in the Parental Role	Emotional Distancing from the Child	Saturation from the Parental Role	Contrast with Previous Parental-Self
Children WO-SN					
Mean	1.48	1.64	1.23	1.19	1.35
SD	0.58	0.71	0.46	0.48	0.56
					
Children W-SND					
Mean	2.12	2.55	1.64	1.48	1.56
SD	0.95	1.19	0.77	0.73	0.70
					
t	7.80	8.67	6.09	4.46	3.16
*p*	0.000	0.000	0.000	0.000	0.010
Effect Size					
(Cohen’s d)	0.81	0.93	0.65	0.47	0.33

Range: 1–7 (7 = high level of PB).

**Table 2:** Risk factors and protective sources for PB by group (W-SND/WO-SN).

	Risk Factors		Protective Resources		
	Perceived Caregiver Burden	Child Functioning	Social Support	Learned Resourcefulness	Deep Emotion Work
Range	1–7	(standardized)	1–5	1–6	1–5
Children WO-SN					
Mean	2.15	−0.01	2.60	4.36	2.39
SD	0.91	0.88	0.91	0.77	0.93
					
Children W-SND					
Mean	2.77	0.01	2.53	4.14	2.99
SD	1.07	0.91	0.91	0.69	0.72
					
t	5.79	0.24	0.74	2.77	6.77
p	0.000	0.086	0.470	0.035	0.000
Effect Size					
(Cohen’s d)	0.62	0.02	0.08	0.30	0.72

## Data Availability

The original contributions presented in the study are included in the article. Further inquiries can be directed to the corresponding author.
